# Associations Between Personality Traits and Longitudinal Change in Physical Function in Survivors of Childhood Cancer

**DOI:** 10.1002/cam4.70965

**Published:** 2025-05-09

**Authors:** Megan E. Ware, Matthew D. Wogksch, Michael Neel, Robyn E. Partin, Jennifer Q. Lanctot, Daniel A. Mulrooney, Melissa M. Hudson, Kirsten K. Ness

**Affiliations:** ^1^ Department of Kinesiology, Health Promotion, and Recreation University of North Texas Denton Texas USA; ^2^ Department of Epidemiology and Cancer Control St. Jude Children's Research Hospital Memphis Tennessee USA; ^3^ Department of Hematology St. Jude Children's Research Hospital Memphis Tennessee USA; ^4^ Department of Surgery St. Jude Children's Research Hospital Memphis Tennessee USA; ^5^ Cancer Center Administration St. Jude Children's Research Hospital Memphis Tennessee USA; ^6^ Department of Oncology St. Jude Children's Research Hospital Memphis Tennessee USA

**Keywords:** childhood cancer survivors, exercise intervention, follow‐up, personality traits

## Abstract

**Objective:**

Exercise interventions for survivors of childhood cancer (survivors) focus on improving cardiovascular fitness and muscle strength. We aimed to examine associations between personality traits and change in physical function outcomes in survivors who completed a resistance training (RT) intervention.

**Methods:**

Participants were 5+ year survivors in the St. Jude Lifetime Cohort. Personality traits were assessed using the Big Five Inventory 2 (BFI‐2). Grip strength was measured using hand‐held dynamometry. Quadriceps strength was measured using isokinetic dynamometry. Walking speed was measured with the Six‐Minute Walk Test (6MWT). Associations between personality traits and grip strength, quadriceps strength, and walking speed were evaluated using multivariable regression adjusted for gender, age at assessment, race, primary cancer diagnosis, and grade 3‐4 chronic health conditions (National Cancer Institute Common Terminology Criteria for Adverse Events, version 4.03).

**Results:**

Among 43 survivors (mean age 34.6 ± 7.0 years, 55.8% female, 67.4% white, 41.9% leukemia/lymphoma, 3.84 ± 2.02 years post‐intervention), mean trait scores were: agreeableness 4.2 ± 0.5, conscientiousness 3.9 ± 0.7, extraversion 3.4 ± 0.7, negative emotionality 2.6 ± 0.9, and open‐mindedness 3.9 ± 0.7. Mean change post‐intervention to follow‐up in grip strength was −2.1 ± 11.9 kg, quadriceps strength −25.5 ± 27.2 Nm/kg, and walking speed 2.5 ± 14.0 m per minute. Survivors who scored higher in conscientiousness had greater positive change in walking speed from post‐intervention to follow‐up (*β* = 11.35, SE = 3.50, *p* = 0.003).

**Conclusion:**

Personality traits may impact the maintenance of physical function in survivors past intervention windows. Further interventions should consider personality and potentially tailor follow‐up to preserve functioning.

## Graphical abstract

Exercise interventions for survivors of childhood cancer focus on improving cardiovascular fitness and muscle strength but often do not consider personality factors that influence longitudinal maintenance of function. In this study of longitudinal performance change after participation in exercise intervention in survivors, associations were observed between higher levels of conscientiousness and improvement in walking speed ~4 years post‐exercise intervention.

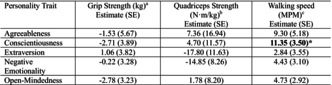

## 1 Introduction

Because of advances in diagnosis and treatment techniques, survival from childhood cancer has improved over recent decades [1]. However, childhood cancer survivors (survivors) are at risk for chronic disease due to cancer or its treatment, which have physiological and psychological consequences such as impaired cardiopulmonary fitness [2], obesity [3], low lean muscle mass [4, 5, 6, 7, 8, 9, 10, 11], muscle weakness [6, 11, 12, 13, 14], and fatigue [15, 16]. While many of these conditions are associated with treatment exposures [17, 18, 19, 20], lifestyle and health behaviors can impact the magnitude of adverse health outcomes after treatment [21, 22, 23]. Therefore, development and implementation of exercise interventions to improve health outcomes and quality of life in this population have become imperative [24].

Personality has been conceptualized as observable, stable traits that (1) have consistent individual‐level differences, and (2) tend to show distinct patterns of thought, feeling, and action [25]. Several conceptualizations of personality have been developed, with a variety of the most popular conceptualization of trait taxonomy is the five factor taxonomy: agreeableness (tendency to be cooperative, altruistic, trustworthy, generous), extraversion (tendency to be sociable, energetic, seek excitement, assertive), neuroticism (tendency to be anxious, self‐conscious, emotionally unstable), conscientiousness (tendency to be ordered, dutiful, self‐disciplined, achievement‐oriented), and openness (tendency to be creative, reflective, perceptive) [26, 27, 28, 29]. Each trait is theorized to have hierarchical relationships with varying levels of constituent facets, or unique aspects or parts of the larger trait [30]. Together, these factors make up a basic personality structure, which is somewhat heritable [31] but also subject to growth and change through the development of sub‐traits based on interactions with individual environments [30], purposeful intervention [32] or as a possible effect of treatment for psychological distress [33]. In the general population, personality traits have been associated with health outcomes, and several theories have been developed to describe potential mechanisms for personality's influence on health outcomes [34]. In survivors, some studies have demonstrated associations between personality traits and health outcomes. Personality trait neuroticism has been associated with young age at sampling, poor self‐rated health, chronic fatigue, and increased depression in long‐term survivors of childhood, adolescence, and young adult cancers [35]. In children with cancer, endorsing depression, anxiety, and post‐traumatic stress symptoms has been associated with neuroticism and negatively associated with all other personality traits from the Big Five Inventory for children [36]. These studies demonstrate the impact of personality on health‐related outcomes particularly in children with and adults who have survived childhood cancer.

Personality has also been hypothesized to moderate the ways in which an individual responds to environmental or internal stimuli, making it an influencing factor on behavior [37]. For this reason, several studies have focused on personality traits and health outcomes and behaviors, leading to meta‐analyses on the topic. Positive cross‐sectional associations have been reported between physical activity and extraversion and conscientiousness, and negative cross‐sectional associations between physical activity and neuroticism [38, 39]. Longitudinal associations between personality traits and physical activity echo associations seen in cross‐sectional analyses, with several studies demonstrating an increased effect of conscientiousness on physical activity across varying lengths of follow‐up [38, 40]. Expanding on these findings, in one analysis of 28,000 participants across nine studies, those with higher levels of conscientiousness, extraversion, and openness had low risk of terminating physical activity over a three‐to‐ten‐year period even if they were inactive at baseline. In contrast, those with higher levels of neuroticism were at high risk of terminating physical activity over the three‐to‐ten‐year period [41]. Collectively, these findings indicate personality traits as a signal of physical activity behavior both acutely and longitudinally. Because of the known link between behavior and physical function, these findings also signal a potential role of personality traits in function.

Understanding the association between personality traits and physical activity or exercise intervention outcomes in survivors is valuable, as this knowledge informs researchers of characteristics of intervention participants who are more or less likely to adhere to the intervention protocol. For example, a resistance training intervention delivered by Krull et al. [42] reported that resistance training among a cohort of adult survivors of childhood cancer improved muscle strength and walking speed outcomes. Among participants, survivors who had higher negative emotionality personality trait scores were less adherent to the resistance training program (*β* = −6.45, SE = 3.01, *p* = 0.04) [42]. These survivors continue to be followed as part of a larger observational cohort study [43], providing an opportunity to evaluate the impact of personality on the maintenance of performance outcomes several years after the completion of the intervention. Because the maintenance of physical function outcomes could be indicative of physical activity or exercise behavior, identifying traits associated with a decline in performance outcomes could inform researchers of characteristics of survivors who need support beyond the intervention window to maintain exercise behavior. The purpose of this analysis was to examine the association between personality traits and change in physical function outcomes after longitudinal follow‐up.

## 2 Methods

### 2.1 Participants

Participants for this study were selected from a subset of the St. Jude Lifetime (SJLIFE) cohort [43] who participated in an ancillary exercise intervention previously described [42]. The SJLIFE cohort is comprised of survivors of childhood cancer who were treated at St. Jude Children's Research Hospital (SJCRH) between 1962 and 2012 and survived 5 years or more since diagnosis [43]. The aim of the SJLIFE study is to track long‐term health outcomes in these survivors. Briefly, participants in the exercise intervention (*n* = 57) were recruited from the SJLIFE cohort to test the effectiveness of protein supplementation with resistance training to improve lean body mass, muscle strength, walking speed, self‐reported exhaustion, and physical activity. The resistance training regimen delivered to both groups consisted of three resistance training sessions per week for 24 weeks with tapered supervision (2 times per week for the first 4 weeks, 1 time per week for weeks 5–12, every other week for weeks 13–20, and 1 time in the last month of intervention). The intervention group in this study received the protein supplementation along with resistance training while the control group received just resistance training. Overall completion of the intervention was 61.3% [42]. Those who had (1) completed a personality assessment at the end of the exercise intervention (hereafter referred to as post‐intervention) and (2) returned to St. Jude Children's Research Hospital for a SJLIFE cohort evaluation after the post‐intervention visit (hereafter referred to as follow‐up visit) were eligible for inclusion in the present analyses. Average time between post‐intervention and follow‐up visit varied and was calculated in final analyses. Informed consent was obtained from all participants prior to participation, and the study was approved by the SJCRH Institutional Review Board (#00000029 FWA00004775).

### 2.2 Measures

#### 2.2.1 Personality Traits

Personality traits were assessed using the Big Five Inventory‐2 (BFI‐2) [44] post‐intervention. The BFI‐2 is a 60‐item assessment that asks participants to respond with how true they feel the items are about themselves on a Likert scale from 1 (“disagree”) to 5 (“Agree”). These responses are scored to quantify five levels of personality traits: extraversion, negative emotionality (neuroticism), agreeableness, conscientiousness, and openness to experience. Scores from each domain range from 1.0 to 5.0 [44]. Continuous scores for each domain were used for the present analyses.

#### 2.2.2 Hand Grip Strength

Isometric handgrip strength was measured while participants were seated. Participants were guided to position the shoulder in 0°–10° of flexion, the elbow in 90° of flexion, and the forearm in neutral using a Baseline handheld dynamometer (Fabrication Enterprises Inc., White Plaines, NY) [45]. The average of two measurements from each hand was used to determine body mass index (BMI)‐specific strength values (kg) [46], a more comprehensive indicator of muscle strength capacity within a larger population where sub‐samples vary in body size. The difference in grip strength was calculated by subtracting the SJLIFE follow‐up visit grip strength value from the post‐intervention visit grip strength value. This difference was used as the grip strength outcome in analyses.

#### 2.2.3 Quadriceps Strength

Isokinetic quadriceps strength was measured in sitting using a Biodex System III dynamometer (Biodex Medical Systems, Shirley, NY) capturing peak torque (N·m/kg) from five repetitions at 60° per second [47]. The difference in quadriceps strength was calculated by subtracting the SJLIFE follow‐up visit value from the post‐intervention visit value. This difference was used as the quadriceps strength outcome in analyses.

#### 2.2.4 Walking Speed

Walking speed was evaluated using the Six‐Minute Walk Test (6MWT) [48]. Participants were instructed to walk as fast as possible while maintaining balance and safety for 6 min. The overall walking distance was recorded in meters. The distance in meters walked per minute was calculated from the overall 6MWT distance/6 (MPM). The difference in MPM was calculated by subtracting the SJLIFE follow‐up visit value from the post‐intervention visit value. This difference was used as the walking speed outcome in analyses.

#### 2.2.5 Demographic and Clinical Characteristics

All demographic and clinical data were evaluated at the post‐intervention visit. Age, sex, and race were obtained via self‐report. Primary diagnosis and treatment information were obtained from medical records. The presence and severity of chronic conditions were assessed at the post‐intervention visit. Chronic conditions were graded according to SJLIFE modified National Cancer Institute (NCI) Common Terminology Criteria for Adverse Events (CTCAE) criteria [49]. Randomization assignment in the exercise intervention was documented for each participant.

#### 2.2.6 Analysis

Demographic and clinical characteristics of participants and non‐participants were summarized using descriptive statistics. *T*‐test and chi‐squared or Fisher's exact tests were used to assess differences between participants and non‐participants. Average personality trait domain scores were calculated for participants only. Average change in grip strength, quadriceps strength, and walking speed from post‐intervention to follow‐up were calculated for the sample, the intervention group, and the placebo group. *T*‐test and chi‐squared or Fisher's exact tests were used to assess differences in change in grip strength, quadriceps strength, and walking speed between the intervention group and the placebo group.

Multivariable linear regression models were used to evaluate associations between continuous personality trait scores and changes in grip strength, quadriceps strength, and walking speed from post‐intervention to follow‐up in the whole sample. Age at post‐intervention assessment, sex, race, diagnosis group, and presence of CTCAE grades 3+ chronic health conditions were a priori selected for inclusion in the models. In addition, each of the models was adjusted for post‐intervention grip strength, quadriceps strength, and walking speed values, respectively. In whole group analyses, randomization assignment was included as a covariate. Analyses were conducted using SAS 9.4 (Statistical Analysis System [RRID: SCR_008567] ver. 9.4, Cary, NC).

## 3 Results

Forty‐three participants (55.81% female, 67.44% White) were eligible for analysis. Participants differed from non‐participants (those who completed the study intervention but did not (1) complete a personality assessment and/or (2) return for a subsequent SJLIFE visit, *n* = 14) in the distribution of sex (*p* = 0.03). Participants were, on average, 3.8 ± 2.02 years post‐intervention and 34.6 ± 6.97 years of age. Leukemia/lymphoma was the most common cancer diagnosis (41.86%), followed by other malignancies (mostly solid [34.88%] and CNS [23.3%] tumors) (23.26%). Average personality trait scores across the sample were highest in agreeableness (4.22 ± 0.47), followed by open‐mindedness (3.88 ± 0.72), conscientiousness (3.86 ± 0.70), extraversion (3.38 ± 0.72), and negative emotionality (2.64 ± 0.91) (Table 1).

**TABLE 1:** Descriptive Characteristics of the study sample.

Characteristic	Non‐participants (*n* = 14)		Participants (*n* = 43)		*p*
	Mean (SD)	No. (%)	Mean (SD)	No. (%)	
Age at post‐intervention assessment (years)	30.73 (6.52)		34.62 (6.97)		0.83
Sex					
Male		11 (78.57)		19 (44.19)	0.03*
Female		3 (21.43)		24 (55.81)	
Race					
White		9 (64.29)		29 (67.44)	0.13
Black		5 (35.71)		12 (27.91)	
Other		0 (0.00)		2 (4.65)	
Diagnosis group					
Leukemia/lymphoma		6 (42.86)		18 (41.86)	0.08
CNS tumor		3 (21.43)		10 (23.26)	
Other		5 (35.71)		15 (34.88)	
Radiation					
Yes		6 (42.86)		21 (48.84)	0.70
None		8 (57.14)		22 (51.16)	
Chemotherapy					
Yes		9 (64.29)		31 (72.09)	0.74
None		5 (35.71)		12 (27.91)	
Surgery					
Yes		14 (100.00)		42 (97.67)	0.75
None		0 (0.00)		1 (2.33)	
Cardiac condition grade 3+ at post‐intervention assessment					
Yes		4 (28.57)		10 (23.26)	0.73
No		10 (71.43)		33 (76.64)	
Musculoskeletal condition grade 3+ at post‐intervention assessment					
Yes		5 (35.71)		19 (44.19)	0.58
No		9 (64.29)		24 (55.81)	
Pulmonary condition grade 3+ at post‐intervention assessment					
Yes		0 (0.00)		6 (13.95)	0.57
No		14 (100.00)		37 (86.05)	
Personality trait score					
Agreeableness			4.22 (0.47)		
Conscientiousness			3.86 (0.70)		
Extraversion			3.38 (0.72)		
Negative emotionality			2.64 (0.91)		
Open‐mindedness			3.88 (0.72)		
Grip strength (kg)					
Post‐intervention to follow‐up change			−3.84 (11.72)		
Quadriceps strength (N·m/kg)					
Post‐intervention to follow‐up change			−24.62 (31.96)		
Walking speed (MPM)					
Post‐intervention to follow‐up change			2.51 (13.98)		

* Statistical significance at alpha = 0.05.

Average grip strength (kg), quadriceps strength (N·m/kg), and walking speed (MPM) are shown in Table 2. Grip strength and quadriceps strength declined over the longitudinal period −3.84 ± 11.72 kg and −24.62 ± 31.96 N·m/kg, respectively. Walking speed, however, improved marginally (2.51 ± 13.98 MPM). (Table 1).

**TABLE 2:** Linear Regression Results of Personality Traits and Performance Outcomes (*n* = 43).

Personality trait	Grip strength (kg)^a^, estimate (SE)	Quadriceps strength (N·m/kg)^b^, estimate (SE)	Walking speed (MPM)^c^, estimate (SE)
Agreeableness	−1.53 (5.67)	7.36 (16.94)	9.30 (5.18)
Conscientiousness	−2.71 (3.89)	4.70 (11.57)	11.35 (3.50)*
Extraversion	1.06 (3.82)	−17.80 (11.63)	2.84 (3.55)
Negative emotionality	−0.22 (3.28)	−14.85 (8.26)	4.43 (3.10)
Open‐mindedness	−2.78 (3.23)	1.78 (8.20)	4.73 (2.92)

^a^ Model adjusted for: age at post‐intervention assessment, sex, race, diagnosis group, presence of CTCAE grades 3+ cardiovascular, musculoskeletal, and pulmonary conditions, and post‐intervention grip strength (kg).

^b^ Model adjusted for: age at post‐intervention assessment, sex, race, diagnosis group, presence of CTCAE grades 3+ cardiovascular, musculoskeletal, and pulmonary conditions, and post‐intervention quadriceps strength (N·m/kg).

^c^ Model adjusted for: age at post‐intervention assessment, sex, race, diagnosis group, presence of CTCAE grades 3+ cardiovascular, musculoskeletal, and pulmonary conditions, and post‐intervention walking speed (MPM).

* Statistical significance at alpha = 0.05.

After adjustment for sex, age, primary diagnosis group, and CTCAE‐graded chronic conditions, personality trait scores were not associated with change in grip strength from post‐intervention to follow‐up. Similarly, personality trait scores were not associated with change in quadriceps strength from post‐intervention to follow‐up. However, higher scores in conscientiousness were associated with greater positive change in walking speed (*β* = 11.35, SE = 3.50, *p* = 0.003) (Table 2). In this model, female gender (*β* = −11.07, SE = 4.09, *p* = 0.01), faster walking speed post‐intervention (*β* = −0.32, SE = 0.14, *p* = 0.03) and pulmonary conditions (*β* = −21.61, SE = 7.05, *p* = 0.005) were associated with slower walking speed at SJLIFE follow‐up.

## 4 Discussion

This investigation provides evidence of an association between conscientiousness and improved walking speed several years after an exercise intervention in adult survivors of childhood cancer [50]. Additionally, our results are consistent with reports of associations between personality traits and walking speed observed in five large studies, where results indicate that lower neuroticism and higher extraversion, conscientiousness, and openness are predictive of faster walking speed and slower declines [51]. Of note, walking speed in healthy young adults typically does not change between the ages of 18 and 50 years. However, nearly 6% of adult survivors of childhood cancer 18–39 years old have declines in walking speed to values consistent with frail health [52] and a loss of 0.1 m/s is a clinically meaningful in geriatric populations [53]. Thus, both the observed average improvement of walking speed several years after an exercise intervention in this study are encouraging.

Conscientiousness reflects “the propensity to be self‐controlled, responsible to others, hardworking, orderly, and rule‐abiding” [54]. As described by Costa, McCrae, and Dye (1991), the facets of conscientiousness are competence (sense of capability, sensibility, and accomplishment), order (tendency to keep one's space tidy and well‐organized), dutifulness (adherence to perceptions of standards of conduct), achievement striving (need to achieve), self‐discipline (ability to persist in a task despite distraction), and deliberation (tendency to be cautious, thoughtful, and plan) [55]. As previously described, personality can be both biologically‐driven and modifiable; conscientiousness and its facets specifically can be heritable [31], but also can be refined through social exposures during childhood leading to greater self‐regulation [56]. Of note, due to the nature of the childhood cancer experience, it is possible that social and environmental exposures cause changes in conscientiousness and its facets early in life. While conscientiousness has not been investigated in this context, one study investigating occurrence of neuroticism in a sample of long‐term survivors of adolescent and young adult cancers (*n* = 1629, median age 31 [range: 0–39] years at survey, 69% female) reported significant bivariate associations between higher levels of neuroticism in survivors who were diagnosed as children or adolescents when compared to young adults (OR: 1.48, 95% CI 1.19–1.84, *p* < 0.0001). Further, fluctuations or changes in personality throughout the lifespan could also be impacted by this unique survivorship experience.

Many studies have documented the link between conscientiousness and health outcomes, although the causal link between the two is complex [57]. The majority of results in the extant literature indicate that those with higher levels of conscientiousness have a lower frequency of chronic disease [58] and lower physiological health risks [59]. Lack of engagement in risky health behaviors, such as physical inactivity, in favor of optimal health behaviors likely contributes to positive health outcomes among those with higher levels of conscientiousness [59, 60, 61]. Key facets of conscientiousness, such as self‐discipline and responsibility, support those with high levels of conscientiousness in engaging in risk‐adverse behavior [62], even in times of high stress [63]. This could make conscientious individuals more likely to engage in follow‐up and less likely to be noncompliant with the intervention [64]. Notably, current literature does not have a large focus on these outcomes in light of personality traits in childhood cancer survivors. This information could expand on the understanding of risk for chronic disease, by incorporating aspects of personality into the current understanding of treatment‐related, psychological, and behavioral risk factors for chronic disease in this high‐risk population.

Several studies have sought to describe associations between conscientiousness and indicators of fitness and performance. In one such study of 12,188 participants (average age ~67 ± 11 years, 59% female, 14% African American), faster walking speed was associated with higher levels of conscientiousness (standardized *β* coefficient = −0.10, *p* < 0.01), even after adjustment for body mass index (BMI), self‐reported smoking status, and self‐reported moderate intensity physical activity level [65]. This study also examined associations between facets of conscientiousness in a six‐facet model [66] and walking speed. Those with higher levels of self‐control (standardized *β* coefficient = −0.03, *p* < 0.01), order (standardized *β* coefficient = −0.04, *p* < 0.01), traditionalism (standardized *β* coefficient = −0.04, *p* < 0.01), and virtue (standardized *β* coefficient = −0.04, *p* < 0.01) had faster walking speeds than those with lower levels, after adjustment for BMI, self‐reported smoking status, and self‐reported moderate intensity physical activity level. However, after adjustment for trait‐level conscientiousness, only higher levels of the facets industriousness (standardized *β* coefficient = −0.10, *p* < 0.01) and responsibility (standardized *β* coefficient = −0.08, *p* < 0.01) maintained favorable associations with walking speed [65]. In a longitudinal investigation of walking speed in 740 older adults (average age 75.2 ± 2.7 years at baseline assessment, 50% female, 48% African American), higher levels of conscientiousness were associated with higher initial walking speed (*β* = 0.016, *p* < 0.05) and less decline in walking speed over 3 years (*β* = 0.011, *p* < 0.05), even after adjustment for sociodemographic characteristics [67]. Additional analyses of associations between conscientiousness and decline in walking speed adjusting for several clinical factors such as cardiac conditions, diabetes, cognitive function, and BMI yielded similar results [67]. Collectively, these findings, as well as the findings of the present study, suggest protective effects of higher levels of conscientiousness in walking performance across varying populations with varying comorbidities. However, these associations need to be further investigated to form more robust conclusions.

The results of the present study were not indicative of associations between any of the personality traits measured and muscular strength. This finding is not supported by other studies in the extant literature. Positive associations were observed in grip strength and conscientiousness in a sample of 12,188 participants (average age ~67 ± 11 years, 59% female, 14% African American) (standardized β coefficient = 0.5, *p* < 0.01) [65]. Another study of 1220 participants (average age 58 ± 16 years old, 50% male, 66% white) found associations between high levels of neuroticism paired with either low extraversion (*β* estimate = 0.17; *p* = 0.008) or low conscientiousness (*β* estimate = 0.18; *p* = 0.013) and lower quadriceps peak torque [68]. Physical activity level could be influential to the deficits observed in strength in these studies. While these results are pertinent to the current findings, survivors of childhood cancer experience persistent skeletal muscle deficits due to treatment exposures—such as methotrexate and vincristine [69]—that could be exacerbated by lifestyle and mask associations between personality and strength. Future studies in survivors could investigate associations between personality traits and muscular strength to gain understanding of the role of personality in addition to other exposures in muscular strength outcomes within and outside of interventions.

### 4.1 Limitations

The results of this study should be interpreted in light of several limitations. Only a small proportion of the larger SJLIFE cohort participated in this ancillary intervention; this does present limitations regarding the potential for small sample bias. Unfortunately, this is unavoidable due to the retrospective nature of the data analyses; these data were collected as part of an exercise intervention [42]. Therefore, it is possible that these data are influenced by volunteer bias, such that participants with certain traits and capabilities were more likely to volunteer for and participate in the exercise intervention itself and be eligible for these analyses [70]. Evidence in the general population describes the role of conscientiousness in various health‐related outcomes, as well as fitness and performance outcomes (as previously discussed); the results of the present study are in line with these findings. However, because survivors have experiences that potentially could alter or shape personality in ways that individuals in the general population do not, such a conclusion cannot be definitively made. Therefore, future studies should seek to include larger sample sizes to more optimally examine individual personality differences [71]. This sample was largely white, which is reflective of the larger cohort, but not reflective of the diversity in childhood cancer survivor groups. Further studies should seek to recruit more diverse samples. Obtaining objective data on physical activity behavior, such as actigraphy data, including pre‐intervention levels, would have provided more context to the association observed between personality traits and functional outcomes. Additionally, obtaining data on confounders that were not obtained as part of this intervention study, such as psychosocial support or well‐being, could have provided more context to the association observed. Further studies should consider utilizing these methods to provide context surrounding longitudinal follow‐up on physical function in survivors. While personality traits are considered stable, change across the lifespan is possible [72]. Therefore, it is possible that some participants experienced personality change between the post‐intervention and follow‐up timepoints. Future longitudinal studies should consider incorporating multiple measures of personality to track changes throughout the lifespan, or throughout a designated follow‐up period. Finally, typological approaches to investigating associations between personality and performance provide more nuanced information in the general population; given the small sample size in the present study, this approach was not deemed acceptable [73]. Future studies in survivors would benefit from larger samples to be powered to detect typological differences in intervention outcomes.

### 4.2 Clinical Implications

Long‐term maintenance of physical function in survivors requires behavioral change from sedentary habits to physically active habits or maintenance of a physically active lifestyle. Exercise interventions (such as those prescribed by exercise physiologists, behavioral scientists, and rehabilitation professionals) delivered in clinical environments are designed with the hope that the behavior will be maintained outside of intervention windows. The results of this study suggest that survivors with higher levels of conscientiousness might retain or even gain upon physical function improvements sparked during exercise interventions. It is possible that survivors who do not have high levels of conscientiousness need more encouragement or closer follow‐up from exercise, behavioral, or rehabilitation practitioners to ensure that physical function levels do not decline after intervention. Therefore, it might be beneficial for clinicians working in this context to administer tests of personality, particularly the Big‐Five Inventory (BFI) prior to beginning intervention with participants. After assessment, practitioners could be better equipped to identify individuals with lower levels of conscientiousness as those who need tailored follow‐up and support throughout and after intervention. While these measures are not currently included in many electronic health record (EHR) systems, there have been calls for more social and behavioral data to be included in EHR due to the evidence of their contribution to health status [74], as well as support for personality to be considered in screening and counseling for cancer [75].

## 5 Conclusions

Adult survivors who have higher levels of conscientiousness personality trait might be more likely to retain fitness intervention outcomes related to walking performance. Maintenance of this outcome can occur despite chronic conditions known to impact fitness. Future exercise interventions in this population should consider assessing personality throughout the intervention as well as post‐intervention and during follow‐up to better understand long‐term maintenance of functional outcomes.

## Author Contributions

**Megan E. Ware:** conceptualization (lead), formal analysis (lead), writing – original draft (lead), writing – review and editing (lead). **Matthew D. Wogksch:** formal analysis (supporting), writing – review and editing (equal). **Michael Neel:** writing – review and editing (equal). **Robyn E. Partin:** writing – review and editing (equal). **Jennifer Q. Lanctot:** writing – review and editing (equal). **Daniel A. Mulrooney:** writing – review and editing (equal). **Melissa M. Hudson:** funding acquisition (lead), writing – review and editing (equal). **Kirsten K. Ness:** conceptualization (equal), funding acquisition (lead), writing – original draft (equal), writing – review and editing (equal).

## Conflicts of Interest

The authors declare no conflicts of interest.

## Data Availability

The data that support the findings of this study are openly available in Zenodo at https://zenodo.org/.
